# Preparing the lethal hit: interplay between exo- and endocytic pathways in cytotoxic T lymphocytes

**DOI:** 10.1007/s00018-016-2350-7

**Published:** 2016-09-01

**Authors:** Hsin-Fang Chang, Hawraa Bzeih, Praneeth Chitirala, Keerthana Ravichandran, Marwa Sleiman, Elmar Krause, Ulrike Hahn, Varsha Pattu, Jens Rettig

**Affiliations:** Cellular Neurophysiology, Center for Integrative Physiology and Molecular Medicine, Saarland University, 66421 Homburg, Germany

**Keywords:** Correlative light and electron microscopy, Early endosomes, Late endosomes, Recycling endosomes, SNARE proteins, Total internal reflection fluorescence microscopy

## Abstract

Cytotoxic T lymphocytes patrol our body in search for infected cells which they kill through the release of cytotoxic substances contained in cytotoxic granules. The fusion of cytotoxic granules occurs at a specially formed contact site, the immunological synapse, and is tightly controlled to ensure specificity. In this review, we discuss the contribution of two intracellular compartments, endosomes and cytotoxic granules, to the formation, function and disassembly of the immunological synapse. We highlight a recently proposed sequential process of fusion events at the IS upon target cell recognition. First, recycling endosomes fuse with the plasma membrane to deliver cargo required for the docking of cytotoxic granules. Second, cytotoxic granules arrive and fuse upon docking in a SNARE-dependent manner. Following fusion, membrane components of the cytotoxic granule are retrieved through endocytosis to ensure the fast, efficient serial killing of target cells that is characteristic of cytotoxic T lymphocytes.

## Introduction

Cytotoxic T lymphocytes (CTL) are a central part of the cellular immune system. They recognize and kill infected cells of the body through directed release of cytotoxic substances. By their T cell receptor (TCR) they specifically recognize pMHC1 molecules on target cells carrying a cognate antigen. This highly specific interaction initiates the formation of an immunological synapse (IS) between the two cells and activates a signaling cascade that results in a series of cellular events ultimately leading to target cell death. The molecular trigger for IS formation involves a protein/protein interaction between the CTL adhesion molecule LFA-1 (Lymphocyte function-associated antigen 1) and the target cell membrane protein ICAM-1 (intercellular-adhesion-molecule 1). Into that region of interaction all proteins are recruited and organized in a so-called SMAC (supra-molecular activation cluster), which is essentially needed for the killing process. The organization of the SMAC involves polarization of proteins, cytoskeleton and organelles towards the IS. A microtubular network is established that transports cytotoxic granules (CG), containing cytotoxic substances such as granzymes and perforin, towards the IS. Finally, fusion of the CG with the plasma membrane occurs, thereby releasing cytotoxic substances into the cleft between CTL and target cell underneath the central SMAC. These substances diffuse to the plasma membrane of the target cell and induce apoptosis. Genetic defects leading to impaired CTL function result in life-threatening diseases like hemophagocytic lymphohistiocytosis, Griscelli syndrome 2, Chediak–Higashi syndrome and many others [1, 2], underlining the importance of this process.

All above processes are described in detail by recent reviews, which the reader may refer to [3–5]. The present review summarizes intracellular vesicle trafficking events that provide the transfer of all necessary molecular components to the IS. We also highlight the process of granule retrieval (endocytosis) after fusion and its significance to CTL function in killing multiple target cells (serial or simultaneous) [6]. A prerequisite of serial killing is the highly synchronized delivery of proteins at the IS which are needed for the fusion process as well as a synchronized endocytosis and recycling of earlier exocytosed membrane material and the generation and maturation of new CG. Since all these processes are interconnected by the endosomal network, in the first part of the review we will focus on endosomal pathways in general. In the second and third part of the review, CG maturation and recycling in CTLs will be described in particular.

## Endosomal network

**Fig. 1:**
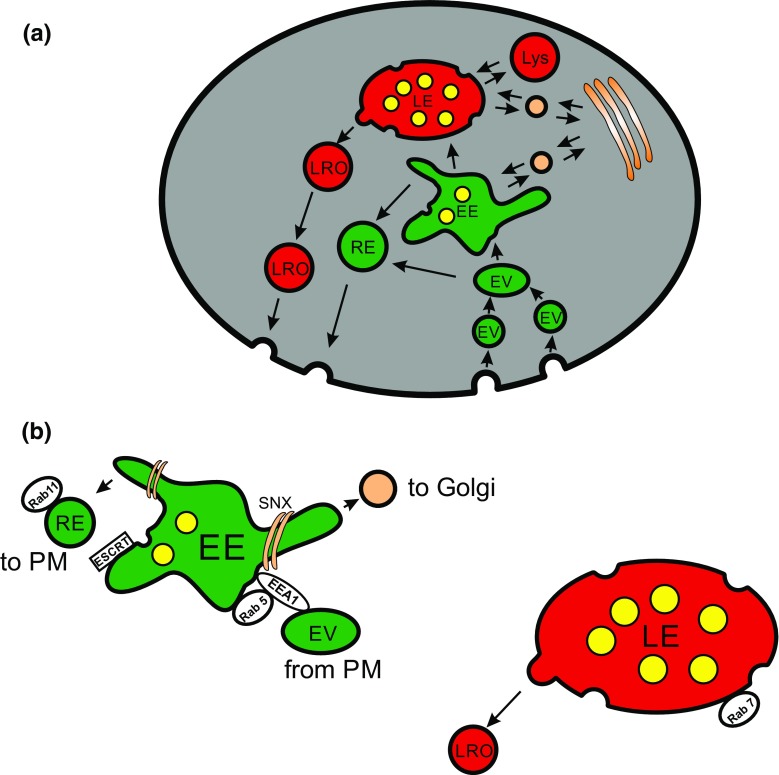
Endosomal pathways. **a** Overview of the major endosomal pathways in mammalian cells. The plasma membrane with protein cargo is endocytosed and forms intracellular endocytic vesicles (EV). Those vesicles homotypically fuse with each other and subsequently fuse with early endosomes (EE). EE are major sorting compartments within the cell. Endocytosed material is sorted into tubular structures and bud off as recycling endosomes (RE) which migrate back to the plasma membrane and exocytose there. Moreover, a process starts in early endosomes which results in the formation of endosomal carrier vesicles (*yellow*). During retro- and anterograde connections to the trans-Golgi endosomal cargo, newly formed proteins are exchanged. By an ongoing production of endosomal carrier vesicles and RE, the EE changes its shape and molecular composition and becomes a late endosome (LE). Finally late endosomes fuse with lysosomes (Lys) in which the remaining cargo mainly localized in endosomal carrier vesicles (multivesicular body) is degraded by hydrolytic enzymes. In some cell types such as cytotoxic T lymphocytes, LE can produce lysosomal-related organelles (LRO) that, as secretory lysosomes, become released by regulated exocytosis. Specific cargo may be inserted into LRO via a transport pathway from trans-Golgi via LE to LRO. **b** Closeup of EE and LE with some important molecular components; early endosomal antigen 1 (EEA1), endosomal sorting complexes required for transport (ESCRT), small GTPases (Rab5, Rab7, Rab11), sorting nexin (SNX)

The endosomal network is a complicated and still not fully understood pool of intracellular compartments and vesicles. Membrane material and extracellular cargo are taken up by both clathrin-dependent and clathrin-independent endocytosis and give rise to endocytic vesicles. These vesicles fuse with early endosomes (EE) and mature into late endosomes (LE). During this process recycling endosomes bud off from EE and the remaining LE fuse with lysosomes. Moreover, newly synthesized material from the Golgi apparatus is fed into the endosomal network and vice versa (Fig. 1; [7–9]) to supply enzymes, membrane receptors and necessary membrane material to different endosomes (Fig. 1).

Over the last two decades, knowledge about the function and regulation of endosomes has substantially increased. It was found that an endosomal network exists, that not only degrades endocytosed material through fusion with lysosomes, but also recycles cargo-receptors back to the membrane via recycling endosomes. In hematopoietic cells such as CTLs, even newly generated secretory vesicles (secretory lysosomes [10]) can bud off the Golgi network and are filled with cargo such as perforin or granzymes by connections of the endosomal compartments with the Golgi apparatus [11]. In the following paragraphs, we will describe in detail the different compartments of the endosomal network. For T cell function, the endosomal network is not only interesting because of the mentioned housekeeping functions, but also for the fact that cytotoxic granules as secretory lysosomes are produced by this network. This makes a detailed understanding of the function and the components of endosomal pathways necessary for understanding immune function.

### Early/sorting endosomes

The first endocytic compartment, which accepts incoming cargo internalized at the plasma membrane, is the early endosome (EE), sometimes also called the sorting endosome. It was already shown that newly formed endocytic vesicles undergo homotypic fusion or they fuse with preexisting EE in a process that requires SNARE and Rab (Ras-associated binding) proteins [12]. The most extensively investigated protein of the Rab family is Rab5. It is attributed to many different functions in early endocytic events. Rab5 can bind and activate the PtdIns3P-kinase with a primary role of generating PtdIns3P (phosphatidyl-inositol-3-phosphate), the most abundant phosphoinositide in the membrane of the EE [13, 14]. Another interacting partner of Rab5 is the early endosomal antigen 1 (EEA1), the most important protein related to the process of endocytic membrane docking and fusion at the EE. It is thought to be exclusively localized on the EE [15]. The dual binding of EEA1 to Rab5 and PtdIns3P tunes its localization to EE membranes [16], and its interaction with an endosomal SNARE complex makes it absolutely essential for the EE fusion in vivo [17]. Meanwhile, more proteins of the Rab family are found to be located on the EE, namely Rab10, Rab14, Rab21, and Rab22 [18].

A new approach to identify EE-specific proteins was published by Duclos and coworkers [19]. They succeeded in isolating early and late endosomes/lysosomes in macrophages and late endosomes/lysosomes in immature and mature dendritic cells and investigated the protein content of these organelles by mass spectrometry. The most challenging step in this procedure was to reach a sufficiently high degree of purification of these endocytic organelles to perform reliable proteomics analysis. They found that most of the Rab proteins such as Rab11a, Rab21, Rab22a, Rab14 Rab1a and Rab5 are localized on EEs.

Depending on its respective function, a cargo protein present in an EE can be directed to three different destinations: the lysosome for degradation, the trans-Golgi network (TGN) or the cell surface via RE. The sorting of membrane proteins to multiple destinations requires an acidic luminal pH (pH ~6.0) that causes the ligands to dissociate from their receptors [20]. After that step a specialized machinery recognizes the cargo proteins and partitions them into discrete domains within the EE, thereby preparing the delivery to the appropriate destination. These processes are associated with morphological changes of the early endosome [21]. Electron microscopy images revealed that the EE is a dynamic compartment with a high homotypic fusion capacity and at least two functionally distinct and separate microdomains [11], namely cisternal regions or thin tubules (~60 nm diameter, several hundred nm length) and large internal vesicles (~300–400 nm diameter) [22]. In general, it appears that cargo located after redistribution in tubules will be recycled to the plasma membrane via RE while the content of internal vesicles (endosomal carrier vesicles—ECV) will undergo degradation [8], preferably after the EE has matured into an LE. In case of recycling of receptors from EE back to other membranes it was shown that the formation of comprehensive endosomal tubular structures facilitates this process [12] and that actin and intact microtubules are required for both endosomal tubulation and fission [9, 23].

The transport of cargo from EE to the TGN [24] mediates the retrieval of various transmembrane receptors, such as the cation-independent mannose 6-phosphate receptor [25]. It is initialized by a retromer machinery which is preferentially recruited to maturing EEs containing increasing concentrations of PtdIns(3,5)P2 generated by PIKfyve kinase [26] and an increased number of intraluminal vesicles [27], both hallmarks of maturation into late endosomes.

The intraluminal vesicles themselves are part of the degradative function of EE. To build up intraluminal vesicles, the cytosolic domains of endocytosed receptors become conjugated by ubiquitin on flat clathrin lattices that are present only on selected EE membranes. As a next step proteins of the “endosomal sorting complexes required for transport” (ESCRT 0-III) will bind to endosomal membranes in a sequential manner. First, ESCRT-0 binds to PtdIns3P of the EE membrane and clusters the ubiquitinylated proteins by multiple binding sites. ESCRT-I and ESCRT-II arrive and bind to the complex by interacting with each other, the cargo and the membrane. Finally, ESCRT-III binds to a subunit of ESCRT-II (VPS25) and initiates the inward vesiculation at the limiting membrane of the sorting endosome [28]. The production of intraluminal vesicles from EE and also LE is an ongoing process, resulting in so-called multivesicular bodies (MVB) [29]. The sorting of MHC class II molecules within the EE for example is triggered by such mechanisms [30].

As the shape of an EE changes and the number of internal vesicles increases during maturation into LE, its pH further decreases to pH values of 5.5–4.5. Moreover, it actively migrates within a cell. Early endosomes have been shown to move centripetally to the juxtanuclear position following endocytosis of cargo at the cell periphery. Attached motor proteins regulate their complex motion, the interaction with other endosomes and organelles and with the associated microtubule network [9].

### Recycling endosomes

As mentioned in the previous section, many types of cargo that are endocytosed and collected in EE are sorted into tubular structures of the EE. After sorting, vesicular structures bud off from the EE and migrate as recycling endosomes (RE) towards the plasma membrane to incorporate still functional receptors and other integral membrane proteins back into the plasma membrane [9, 31, 32]. For many, if not all cell types, from a quantitative point of view, the recycling endosome pathway is the major route of vesicle trafficking [7, 33], emphasizing its importance for maintaining plasma membrane homeostasis and, therefore, cellular function.

The cargo transported via this pathway is manifold such as LDL receptors [34], MHC receptors [35], CD3 receptors [36, 37] or transferrin receptors [38]. In addition, solutes, SNARE and SNARE-associated proteins and G-protein coupled receptors are also recycled via RE. It can be assumed that almost every reusable cargo undergoes recycling via RE, sometimes with a (currently not fully understood) switch between recycling pathways and degradation [39, 40].

Typical for recycling endosomes is the presence of Rab11 as a marker protein [41]. For fission from EE several proteins like sorting nexins (SNX) [42] and dynamin [43] are necessary. SNARE proteins like VAMP8 may drive fusion of RE at the plasma membrane [44]. The pH of RE is around 6.5 and, therefore, slightly higher as in EE, probably because of the lack of v-ATPases [45].

Aside from RE, another group of vesicles also participates in the recycling processes. These recycling vesicles constitute the so-called fast or rapid recycling [9]. They are rapidly produced after endocytosis—either shortly after fusion to EE or even before. In contrast, the classical RE is produced later after an EE has reached a position deep into the cell near the MTOC (slow recycling).

For fast recycling, Rab4 [46] and Rab35 [47] appear to be important, though their function is not entirely clear. Examples of cargo going through the fast recycling pathway might include the transferrin receptor [48] or membrane lipids [49]. Since the fast recycling vesicles do not possess Rab11 (a classical RE marker) and are not a product of classical EE sorting, they do not belong to the group of RE.

### Late endosomes

There is ongoing debate about the exact step at which an EE becomes an LE, because the transition of EE to LE is a continuous process driven by fission and fusion processes which gradually change the character of endosomes [50]. Therefore, the definition is mostly operational and does not necessarily reflect a different function. For example, both subpopulations can exchange material with the Golgi and can send vesicles for exocytosis to the plasma membrane [51]. Most authors agree that an EE is characterized by two marker proteins EEA1 and Rab5 [13, 52] which are missing in LE. LE or related organelles are in contrast characterized by the presence of Rab7 [53]. In contrast to EE, the pH in LE is decreased substantially to values between 5.0 and 4.5.

A mature LE has morphologically lost its tubular structures and possesses a huge number of intraluminal vesicles. The membrane of those vesicles contains proteins that are endocytosed from the plasma membrane and intended for degradation. In contrast, most material that is intended for recycling to the PM might not be present. The fate of a mature LE is unidirectional in its progress and will eventually fuse with a lysosome and become itself a lysosome. Decisive for the fusion process is Rab7 and an effector complex which is termed HOPS in yeast [54, 55]. In mammals, it is probably composed of different SNAREs and SNARE-associated proteins [56].

### Lysosomes

Lysosomes are characterized by their acidic pH (pH 4.5–4.0) and a high concentration of proteases, nucleases and lipases. Accordingly, their function is hydrolytic degradation of all content that is delivered by late endosomes. The lysosomal-associated proteins LAMP-1 and LAMP-2, which constitute 50 % of the total lysosomal membrane protein, are useful for identification [57].

A specialized subspecies of lysosomes are secretory lysosomes, which can be found in hematopoietic cells [10]. As the name implies, those lysosomes are intended to undergo regulated exocytosis. Cytotoxic granules in T lymphocytes belong to this group and will be described in the next section.

## Cytotoxic granules

Cytotoxic granules (CG) are the primary effector organelles of CTLs. They undergo exocytosis at the CTL: target cell interface called the immunological synapse (IS) to release their cytotoxic contents such as perforin, granzymes and granulysin which all induce target cell death. CGs are believed to be hybrid organelles having properties of conventional lysosomes and secretory granules and for this reason are referred to as secretory lysosomes or lysosomal-related organelles. This classification is made because CG-specific markers such as perforin and granzyme co-localize with conventional lysosomal markers such as LAMP-1 and LAMP-2, lysosomal transmembrane proteins such as CD63, soluble proteins such as cathepsins and other lysosomal hydrolases such as alpha-glucosidases and acid phosphatases, clearly demonstrating a lysosomal origin of CGs [58, 59].

### Composition, structure and function of CGs

Secretory lysosomes in CTLs show a heterogeneous appearance in electron micrographs varying not only in size (300–700 nm), but also in the amount of electron-dense matrix [60]. Antibody labeling of ultrathin cryosections revealed that there are many classes of mature secretory lysosomes that might represent intermediate endosomal steps of CG maturation. Owing to this variability in the lyso-/endosomal nature of CGs, specific marker proteins cannot be used to define the intermediate stages since they may also be located on diverse vesicles of the endosomal network. As a matter of fact, even the presence of perforin and granzymes alone, the specific effector proteins of CGs, cannot be used to define mature CGs because they are also present in other endosomal compartments such as LE and multivesicular bodies albeit in different amounts, therefore, representing different maturation stages. Despite the lack of a clear CG marker, it is believed that among the heterogenous vesicles identified in CTLs, those packed with an electron-dense proteoglycan core are most probably the most mature CGs [61]. This interpretation is strengthened by our own research, where we used the v-SNARE responsible for final CG fusion at the IS, synaptobrevin2, as a CG marker in correlative light and electron microscopy (CLEM) [60, 62]. Adding to the complexity, however, is the possibility that more than one population of mature CGs exist. Schmidt and coworkers [63, 64] demonstrated, by combining density gradient centrifugation, proteomics and electron microscopy that two subpopulations of CGs exist. Proteomic profiling of T cell organelles separated by density gradient centrifugation revealed the presence of two species of lysosome-related organelles: a larger, electron-light clear fraction with a diameter between 300 and 700 nm and a smaller, electron-dense dark fraction with a diameter of less than 300 nm. The larger fraction was enriched in FasL and classical lysosome makers such as LAMP-1, CD63 and cathepsinD, while the smaller fraction contained cytotoxic effector molecules such as granzymes, perforin and granulysin. The two populations still share 70 % of approximately 400 proteins found. Schmidt and coworkers conclude that one population induces target cell membrane attack via perforin and granzymes and the second population induces target cell apoptosis via activation of target-cell Fas-receptors. This study contradicts other studies that have demonstrated co-localization of FasL with perforin/granzymes [65], adding ambiguity to the ultrastructural identity of mature CGs.

Since CGs are the effector organelles of CTLs, during infection they are transported along the microtubular network of the CTL towards the IS where a low number of CGs (1–5) fuse with the plasma membrane to release their content and kill the infected target cell [66]. Fusion is driven by several SNARE proteins from which synaptobrevin2 was identified as the vesicular SNARE [62] and syntaxin11 [67] as a target SNARE on the plasma membrane. In addition to SNARE proteins, SNARE-associated proteins like Munc18-2 function as a binding partner for syntaxin11 [68]. Rab27a and Munc13-4 are the molecules required for the essential CG pre-fusion steps such as docking and priming, respectively [69–71].

### Maturation of CGs

**Fig. 2:**
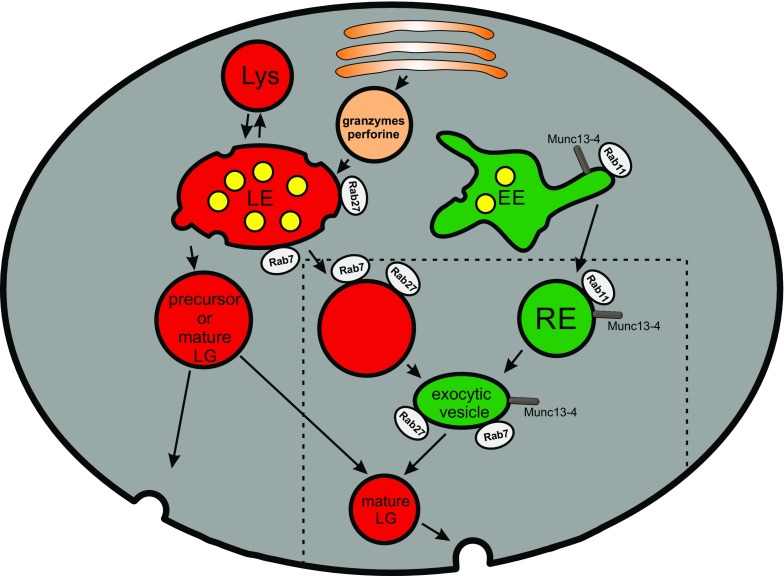
Maturation of cytotoxic granules. Maturation of cytotoxic granules (CG) starts at late endosomes (LE) by budding off of secretory lysosomes (here named “precursor or mature CG”). Granzymes are incorporated into this vesicle by an anterograde transport from the Golgi network to LE and finally to CG. How other components like perforin and membrane effector proteins are incorporated is largely unknown. A new and interesting hypothesis (components inside the *dashed area*) emphasizes a function of recycling endosomes (RE) for maturation of CG. Two additional fusion events were proposed. The first fusion occurs between an LE-derived vesicle carrying different Rab proteins (Rab7, Rab27) and other CG cargo and an EE-derived RE carrying Rab11 and Munc13-4. This fusion, which itself is Munc13-4 dependent, results in an intermediate named exocytic vesicle. This vesicle may then fuse with the LE-derived precursor CG to form a mature CG. *Lys* lysosome

Cytotoxic granules (CGs) are formed from precursor organelles through a series of membrane transport steps (involving TGN and endosomes). The cytotoxic effector molecules perforin and granzymes are transported from the trans-Golgi network to budding endosomal vesicles that are possibly early endosomes. Granzyme A and B are translated into protein at the rough endoplasmic reticulum and mature and are targeted to the Golgi apparatus where both bind to mannose-6-phosphate receptors [72]. Luminal vesicles containing granzymes as cargo bud off from the trans-Golgi aided by the ESCRT complex (endosomal sorting complex required for transport). The luminal vesicles eventually mature by accumulation of cytotoxic effector proteins into the proteoglycan-rich dense core and mature possibly through EE and LE. Tubular EE or sorting endosomes are capable of segregating different cargo into new vesicles that bud off from the tubular endosomes and gradually mature into LE [9, 31, 32]. One might also hypothesize that the newly formed CGs along with cargo proteins are transported from EE to LE and eventually into new vesicles that bud off from LE. Since the LE bears almost all the lysosomal markers, the lysosomal origin of newly formed CGs may be obtained through LE.

de Saint Basile and coworkers proposed that the biogenesis of mature CGs is a multi-step process. This hypothesis based on work by Ménager and coworkers proposes that Munc13-4 is present on Rab11-positive recycling endosomes and Rab27a on late endosomes. The two organelles fuse to generate an intermediate precursor organelle in a Munc13-4-dependent fusion step. This hypothesis, therefore, emphasizes the function of Munc13-4 not only for CG exocytosis at the plasma membrane, but also for the generation of precursor intermediate exocytic vesicle [73, 74] (Fig. 2). This intermediate precursor exocytic vesicle now carrying effector molecules like Rab27a and Munc13-4 that are absolutely essential for the exocytosis of CGs, may fuse or tether with perforin/granzyme containing immature CGs to render a mature CG. The addition of other CG membrane molecules that are essential for CG fusion at the IS is also not well described.

### Exocytosis and endocytosis of CGs

As outlined above, the maturation of fusogenic CGs is still very controversial. By definition, the most mature CGs must be the ones that fuse with the plasma membrane at the IS. A requirement for that fusion process must be the presence of a vesicular SNARE protein on the CG membrane. Work from our lab has shown that in murine primary CTLs, synaptobrevin2, the most important v-SNARE for neuronal synaptic vesicle exocytosis, performs this function [62]. Interestingly, the above-mentioned CLEM on CTLs derived from synaptobrevin2-knockin mice revealed two surprising pieces of data. First, the synaptobrevin2-positive CGs were very homogeneous in diameter (about 350 nm), indicating that, in contrast to the postulate by Schmidt and coworkers [63, 64], only one class of mature CGs exists. This finding was supported by recent combined total internal reflection fluorescence (TIRF) microscopy and membrane capacitance measurements that determined a homogeneous diameter of fusing CGs of 312 nm [66]. The second surprising finding of the CLEM experiment was that not all dense-core granules with a diameter of about 350 nm were positive for synaptobrevin2. Since synaptobrevin2 is essentially required for the fusion of CGs with the plasma membrane, these data might imply that the synaptobrevin2-negative granules are not CGs. Whether they are precursors of mature, fusogenic CGs or belong to an entirely different class of granules remains to be elucidated. Apparently, the synaptobrevin2-knockin mouse provides an excellent tool to unravel the molecular composition of mature CGs.

**Fig. 3:**
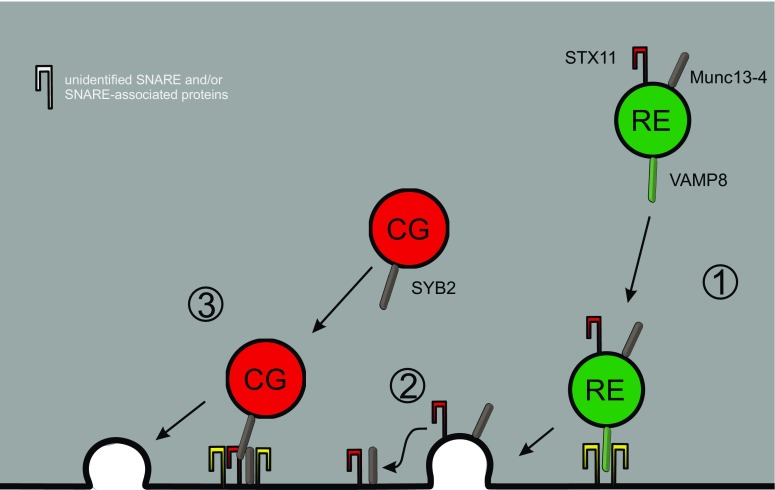
Exocytosis of cytotoxic granules. Secretion of cytotoxic granules (CGs) is a sequential process requiring exocytosis of recycling endosomes (RE) as an initial step (*1*). Thereby REs deliver components of the exocytic machinery for CGs such as the SNARE-associated protein Munc13-4 and the SNARE protein syntaxin11 (STX11) (*2*). Together with further, currently unknown SNARE proteins, those components serve as a docking platform for cytotoxic granules and initiate CG fusion through Munc13-4-catalyzed SNARE complex formation (*3*)

The application of TIRF microscopy also enabled testing of the proposal that Rab27a/Rab11-positive endosomes fuse or tether with cytotoxic granules [73, 74] (Fig. 2). CTLs are plated on coverglass coated with anti-CD3 antibody which results in the formation of an IS at the glass/cell interface. Since the resulting evanescent wave in TIRFM extends only 150 nm into the CTL, labeling of granules with specific markers allows the investigation of granule mobility and fusion with high spatial (and temporal) resolution. TIRFM of CTLs in which RE were labeled with Rab11-GFP and CGs were labeled with granzymeB-mCherry revealed that both vesicle types polarize to the IS and undergo fusion [67]. Importantly, though, their arrival and fusion is sequential, with RE arriving first and CGs arriving and fusing later. This sequential pattern makes sense, because Halimani and coworkers showed that syntaxin11, an essential t-SNARE for CG fusion at the IS, is transported to the IS through RE [67]. The resulting syntaxin11 clusters in the IS plasma membrane then serve as a docking spot for arriving CGs which then form a SNARE complex to mediate fusion and release of their cytotoxic components. Further studies have verified this sequential process and identified VAMP8 as the v-SNARE mediating RE fusion at the IS [44]. Thus, RE do not fuse with CGs, and the tethering of CGs occurs through proteins like Munc13-4 and syntaxin11 that have been transported beforehand to the IS through RE (Fig. 3).

CTLs are serial killers, i.e., they can kill multiple target cells sequentially and efficiently [6, 75, 76]. Therefore, constant generation of fusogenic CGs is required. Though a constant synthesis of new CG components is theoretically possible, a much more efficient way is to retrieve used CG membrane components through endocytosis. It has been shown that essential IS membrane components like the T cell receptor are endocytosed through RE. Therefore, the question arises whether CG membrane components converge with IS plasma membrane components through a joint retrieval through RE. Liu and coworkers tested this hypothesis in NK cells by looking at the retrieval of LAMP-1, the major lysosomal protein that is frequently used in FACS-based degranulation assays to quantify cytotoxic granule release, under different experimental conditions in lipid bilayer-based TIRF microscopy. It was shown previously by FACS that a considerable fraction of LAMP-1 is internalized two hours after contact with target cells [77]. Liu et al. now demonstrated that both LAMP-1 exocytosis and LAMP-1 internalization occurred in a large and spatially stable cluster at the center of the IS. Both processes, as well as the correct spatial organization of receptor–ligand distribution, required the presence of the LFA-1 ligand ICAM-1 [78]. Finally, the authors showed by three-dimensional imaging of fixed cells that perforin-containing CGs were juxtaposed to the LAMP-1 internalization sites, suggesting that the IS contains an LFA-1-dependent area where LG fusion and LAMP-1 internalization occur adjacent to each other. Recently, our laboratory could expand on these finding showing that while LAMP-1 internalization partially overlaps with the endocytosis of cytotoxic membrane components, synaptobrevin2 is a more specific marker protein for CG endocytosis [79]. Key molecules involved in the endocytosis of CG membrane components were dynamin, clathrin and the synaptobrevin2-specific adaptor protein CALM. Importantly, recycling of endocytosed CGs does not include RE, but rather EE and LE. Following refilling with granzyme B at the LE stage, recycled CGs are ready to fuse again and contribute about 50 % to the serial killing of target cells. From these data it can be concluded that CGs are recycled, in contrast to other IS components like TCR and Munc13-4, through a specialized pathway that enters the endosomal pathway not until the EE stage.

In summary, CTLs appear to keep the endosomal pathway and the CG maturation pathway separate to fulfill their physiological function, the selective killing of target cells through controlled exocytosis of CGs at the IS. On the endocytic branch, a specialized internalization pathway for CG membrane components has been developed as well, probably to ensure a fast and efficient recycling during high killing activity.

## Abbreviations

CG: Cytotoxic granule
CLEM: Correlative light and electron microscopy
CTL: Cytotoxic T lymphocyte
EE: Early endosomes
ICAM-1: Intercellular adhesion molecule 1
IS: Immunological synapse
LAMP1: Lysosomal-associated membrane protein 1
LE: Late endosomes
LFA-1: Lymphocyte function-associated antigen 1
PtdIns(3)P: Phosphatidyl-inositol-3-phosphate
pMHC1: Peptide-major histocompatibility complex type 1
Rab: Ras-associated binding proteins
RE: Recycling endosomes
SMAC: Supra-molecular activation cluster
Sybki: Synaptobrevin2-mRFP knockin
Syb2: Synaptobrevin2
SNARE: Soluble *N*-ethylmaleimide-sensitive factor attachment receptor
SIM: Structured illumination microscopy
TCR: T cell receptor
TIRFM: Total internal reflection fluorescence microscopy
TGN: Trans-Golgi network
